# Microplastics in the Baltic Sea region lakes—standardized insights reveal urban shoreline as key driver

**DOI:** 10.1007/s11356-025-37103-x

**Published:** 2025-11-21

**Authors:** Ewa Babkiewicz, Elina Vecmane, Magdalena Fuk, Magdalena Jurgielewicz, Agnieszka Koniuk, Eliza Kurek, Piotr Maszczyk, Magdalena Michalska-Kacymirow, Daiva Jonuskiene, Jolanta Norvaišienė, Valentina Burdukovska, Inta Dimante-Deimantovica, Juris Tunēns, Wojciech Pol, Ewa Bulska

**Affiliations:** 1 University of Warsaw, Biological and Chemical Research Centre, Warsaw, Poland; 2 Univeristy of Warsaw, Faculty of Biology, Department of Hydrobiology, Warsaw, Poland; 3 Latvian Institute of Aquatic Ecology, Riga, Latvia; 4 Foundation for the Protection of Great Masurian Lakes, Giżycko, Poland; 5 Siauliai Chamber of Commerce, Industry and Crafts, Siauliai, Lithuania; 6 Institute of Food Safety, Animal Health and Environment “BIOR”, Riga, Latvia; 7 Region Norrbotten, Gällivare, Sweden

**Keywords:** Microplastics, Freshwater lakes, Shoreline urbanization, Nutrients, Standardized methods, Baltic Sea region

## Abstract

Microplastics (MPs) are ubiquitous in aquatic environments and closely tied to human activities, yet linking measured concentrations to urban pressures remains challenging. Methodological inconsistencies inflate variance, obscure spatial patterns, and hinder comparability. We therefore implemented fully harmonized sampling and FTIR-verified analysis across ten lakes in Latvia, Lithuania, and Poland, with all field and laboratory work performed by the same team. In parallel, we calculated an urbanization index to relate MP concentrations to shoreline development and urban influences. This design enabled comparisons of MPs in surface waters and sediments with ranges of 0.67–7.68 particles L⁻^1^ (water) and 0.09–3.90 particles g⁻^1^ dw (sediments). Polyethylene and polypropylene predominated, with fibres and fragments as the most common shapes; particles < 5 mm, and black, blue, or white, were most frequent. Shoreline urbanization, quantified by the shoreline urbanization index, was the only significant predictor of MPs in surface waters, explaining ~ 74% of variance, while nutrient concentrations, lake type, proximity to point sources, and season showed no consistent effect. Seasonal shifts in particle shape and type were observed but not in total abundance. MP concentrations were broadly similar among countries, with modest, statistically inconclusive differences in particle size and colour, indicating limited regional variation once methodological noise is removed. Our findings highlight that harmonized protocols sharpen the detection of environmental drivers and may overturn previously reported geographic differences driven by inconsistent methods. This first fully standardized, multi-lake, cross-country MP survey provides a robust framework for future monitoring and mitigation in inland waters.

**Supplementary information:**

The online version contains supplementary material available at 10.1007/s11356-025-37103-x.

## Introduction

Plastics, a diverse group of synthetic and semi-synthetic polymers such as polyethylene (PE), polypropylene (PP), polystyrene (PS), and polyhydroxybutyrate (PHB), have seen a steady increase in production and use throughout the twentieth century, resulting in their widespread accumulation in natural environments. Among them, microplastics (MPs), defined as plastic particles smaller than 5 mm, originate both from the fragmentation of larger debris and the intentional use of microscopic particles in consumer products (Campanale et al. 2020). Their pervasive presence in ecosystems, and even in human biological systems, has raised significant environmental and health concerns (Wegener et al. 2014). Numerous studies have documented their toxicological effects, highlighting ingestion across diverse taxa (Roch et al. 2020; Li et al. 2021) and adverse impacts at multiple biological levels, including aquatic organisms and the human food chain (Hartmann et al. 2019; Imhof et al. 2017; Jeong et al. 2017; Li et al. 2022). The extent of harm caused by MPs depends on their physical and chemical properties, such as size, polymer type, density, and additives. MPs exert both direct (e.g., mechanical damage, chemical toxicity) and indirect effects (e.g., pollutant transport, microbial colonization) (Huang et al. 2022). Therefore, robust ecological risk assessment requires integrating biological effects with environmental features such as MP abundance, composition, and distribution; without this context, risk evaluations may misrepresent the true impact of MPs in ecosystems.

Despite increasing evidence of MP harm, research remains limited by the lack of standardized methods, including in freshwater systems. Variability in environmental matrices, particle morphology, sampling goals, and available resources has led to diverse, often incompatible sampling and analytical approaches (Rai et al. 2022; Razeghi et al. 2021; Yin et al. 2023; Earn et al. 2021). Tools, mesh sizes, digestion protocols, and detection technologies vary considerably, and no single method captures all particle types effectively (Sharma et al. 2024; Ivleva 2021). Further challenges arise from differences in terminology, reporting units, and particle classification schemes (Hartmann et al. 2019; Upadhyay and Bajpai 2024), as well as from technical limitations in identifying small or weathered particles (Zhao et al. 2024; Ali et al. 2024). Consequently, reported MP concentrations often diverge by several orders of magnitude, likely due to methodological rather than environmental differences (Koelmans and Kooi 2019). This fragmentation undermines reproducibility, complicates global assessments, and hinders policy development to mitigate pollution (Matavos-Aramyan 2024; Cowger et al. 2020). While several reporting frameworks and best practices have emerged (e.g., Cowger et al. 2020; Zhao et al. 2024), coordinated international efforts are still needed to establish harmonized, cost-effective, and scalable monitoring protocols.

MPs are ubiquitous across freshwater, marine, and terrestrial environments (Morin-Crini et al. 2022; Kumar et al. 2021; Pol et al. 2023). Among freshwater systems, lakes are particularly vulnerable due to their limited water exchange and frequent proximity to urban and industrial centres, which expose them to anthropogenic pollution (Kakade et al. 2021). Functioning as initial inland pollution residence spots, lakes can intercept MPs before these particles move downstream to marine environments, making them crucial for understanding MP transport. Case studies from various regions demonstrate that lakes can act either as net sinks or as sources of MPs. For instance, Lake Geneva retains around 90% of incoming particles, primarily accumulating them in bottom sediments (Boucher et al. 2019), whereas Lake Mjøsa exports 70–90% of its inputs downstream through the river system (Clayer et al. 2021). In contrast, the urban Lake Xinghu in China generally functions as a sink, but during extreme weather events, it can temporarily release previously deposited MPs into outflowing waters (Li et al. 2023). Such variability reflects the unique hydrodynamics and ecological roles of lakes, underscoring the need for targeted MP research.

Scientific interest in MPs in lakes has grown rapidly, as evidenced by at least ten review articles published in 2024 (Chen et al. 2024a, 2024b; Wu et al. 2024). A key finding is the considerable variability in MP abundance and distribution in lakes worldwide, affecting both surface waters and sediments (Yang et al. 2022; Chen et al. 2024a). Based on global lake datasets, Chen et al. (2024a) reported mean MP abundances of 11,742 ± 31,143 items × m^3^ in water and 1072 ± 3102 items × kg⁻^1^ dry weight (DW) in sediments; likewise, for lakes worldwide, Jachimowicz et al. (2026) found 16,002 ± 32,414 MPs × m^3^, with a median of 1000 MPs × m^3^ and a coefficient of variation (CV) of 202% in water, and 3046 ± 7073 MPs × kg⁻^1^ DW, with a median of 226 MPs × kg⁻^1^ DW and a CV of 232% in sediments. This variability is driven by environmental factors (e.g., bathymetry, nutrient levels, spatial heterogeneity, seasonality), anthropogenic influences (e.g., shoreline land-use intensity), and differences in national plastic management policies (Usman et al. 2022). In addition, methodological inconsistencies across studies, encompassing sampling methods, equipment, and analytical protocols, substantially contribute to the reported variation (Yang et al. 2022; Chen et al. 2024a).

Differences in MP concentrations across continents are pronounced, with studies reporting much higher values in Asia (e.g., 42.2 particles × L⁻^1^ in India and 10.8 particles × L⁻^1^ in China) than in Europe (0.4 particles × L⁻^1^) or North America (0.8 particles × L⁻^1^) (Jachimowicz et al. 2026). These differences likely reflect a complex interplay of factors, including waste management practices, industrial activity, population density, and environmental policies; however, discrepancies in sampling methods may also contribute. For example, 52% of studies in China and 85.9% in India relied on simple water samplers such as steel buckets, which typically yield smaller sample volumes and may inflate MP estimates, particularly for floating particles and small fragments. In contrast, European and North American studies more frequently used pumps or manta nets, which can provide larger, more representative samples. Although environmental and socioeconomic drivers influence MP distribution, the role of methodological variation remains insufficiently understood; comparative studies using fully standardized protocols across regions are notably lacking.

Although country-specific factors have been identified as major determinants of MP distributions (Chen et al. 2024a), it remains unclear whether observed differences are primarily driven by environmental variability or methodological inconsistencies (Earn et al. 2021). To directly disentangle these contributions and eliminate procedural bias, the same research team in this study applied standardized sampling and analytical protocols across ten lakes located in Latvia, Lithuania, and Poland, i.e. three European Union countries differing in population density, wastewater treatment infrastructure, industrialization level, and public awareness of plastic pollution (Table 1). These differences, especially in waste management strategies, industrial activities, and population density, could affect baseline MP concentrations and are essential for interpreting spatial patterns observed in our dataset. Given that Poland has a population density three to four times higher than Latvia or Lithuania, along with the highest industrial output, particularly in heavy manufacturing sectors, one might expect Polish lakes to exhibit the highest MP concentrations (Table 1). Another significant difference regarding a possible pollution source is recreational activity in lakes in Poland; due to lake size and traditions, sailing with yachts and related infrastructure is developed in Polish lakes, whereas this is not the case in Latvia and Lithuania. However, available studies from this region do not allow for identification of consistent spatial patterns, either between lakes in different countries or within a single country. This lack of clarity likely reflects three main limitations: first, previous studies have only covered a small number of lakes; second, reported MP concentrations and characteristics vary widely, even among nearby systems; third, sampling often covers only one season or is done without replicates (Barone et al. 2024; Dimante-Deimantovica et al. 2024; Pol et al. 2023; Fojutowski 2024).

**Table 1:** Key characteristics of waste and wastewater management, industrial activity, population pressure, and local policies in Latvia, Lithuania, and Poland that may influence MP characteristics in their lakes

Factor	Latvia	Lithuania	Poland
Population density	~ 29 people/km^2(1)^	~ 45 people/km^2(2)^	~ 124 people/km^2(3)^
Waste management	Relatively high recycling, but limited landfill space^(4)^	Improving waste sorting and recycling^(5)^	Still reliant on landfilling, but recycling is increasing^(6)^
Wastewater treatment	High wastewater treatment, but rural areas lack infrastructure^(7)^	Advanced urban treatment, rural areas remain a challenge^(8)^	Major investments in treatment, but some older systems persist^(9)^
Economic development (GDP per capita)	~ $22,000^(10)^	~ $27,000^(11)^	~ $38,000^(12)^
Industrialization level	Latvia’s industrial activity is relatively modest, with a focus on agriculture, forestry, and low-tech manufacturing sectors such as woodworking and food processing. High-tech industries, including chemicals and pharmaceuticals, contribute significantly to exports but remain less developed compared to other EU nations^(13)^	Moderate industry, balanced with services^(14)^	Highest industrial output, strong in heavy manufacturing^(15)^
Legislation on plastic pollution	EU regulations in place, but enforcement varies^(16)^	Stricter policies being implemented^(17)^	More extensive laws and higher public awareness^(18)^

^1^Central Statistical Bureau of Latvia. (n.d.). Homepage. Retrieved April 2, 2025, from https://www.csp.gov.lv/lv

^2^World Population Review. (n.d.). Lithuania. Retrieved April 2, 2025, from https://worldpopulationreview.com/countries/lithuania

^3^Statistics Poland. (n.d.). Homepage. Retrieved April 2, 2025, from https://stat.gov.pl/en/

^4^Kļavenieks, K. (n.d.). Efficient waste management sector: Summary of the doctoral thesis. Riga: RTU Press

^5^European Environment Agency. (2022). Early warning assessment related to the 2025 targets for municipal waste and packaging waste: Lithuania. Retrieved from https://www.eea.europa.eu/publications/many-eu-member-states/lithuania

^6^Instytut Spraw Publicznych. (2024). The waste sector in Poland. Retrieved from https://www.isp.org.pl/uploads/drive/CEECAW/Waste_Poland_-_wersja_3.pdf

^7^Riga City Council. (2018). Public water management services in Latvia (Operators’ point of view). Retrieved from https://rpr.gov.lv/wp-content/uploads/2018/01/Public_water_mangm_services.pdf

^8^Organisation for Economic Co-operation and Development. (2022). Reform of water supply and wastewater treatment in Lithuania. Retrieved from https://www.oecd.org/en/publications/reform-of-water-supply-and-wastewater-treatment-in-lithuania_f966a980-en.pdf

^9^European Environment Agency. (2022). Poland – Water Information System for Europe. Retrieved from https://water.europa.eu/freshwater/countries/uwwt/poland

^10^CEIC Data. (2022). Latvia GDP per capita. Retrieved from https://www.ceicdata.com/en/indicator/latvia/gdp-per-capita

^11^Trading Economics. (2023). Lithuania GDP *per capita*. Retrieved from https://tradingeconomics.com/lithuania/gdp-per-capita

^12^Macrotrends. (2025). Poland GDP per capita 1990–2025. Retrieved from https://www.macrotrends.net/global-metrics/countries/pol/poland/gdp-per-capita

^13^Britannica. (2024). Latvia – Economy, agriculture, industry. Retrieved from https://www.britannica.com/place/Latvia/Economy

^14^Lloyds Bank Trade. (2025). The economic context of Lithuania – International trade portal. Retrieved from https://www.lloydsbanktrade.com/en/market-potential/lithuania/economical-context

^15^WorldAtlas. (n.d.). The biggest industries in Poland. Retrieved from https://www.worldatlas.com/articles/the-biggest-industries-in-poland.html

^16^Ministry of Environmental Protection and Regional Development. (2023). Restriction of plastic products will reduce environmental pollution and improve waste management. Retrieved from https://www.varam.gov.lv/en/article/varam-restriction-plastic-products-will-reduce-environmental-pollution-and-improve-waste-management

^17^European Environment Agency. (2022). Lithuania – European Environment Agency. Retrieved from https://www.eea.europa.eu/publications/many-eu-member-states/lithuania

^18^CMS Law. (2024). Plastics and packaging laws in Poland. Retrieved from https://cms.law/en/int/expert-guides/plastics-and-packaging-laws/poland

Notably, the high variability observed in earlier studies, despite comparable regional and climatic conditions, suggests that methodological differences may be a significant source of inconsistency, potentially overshadowing true environmental variation. By using a single research team, identical equipment, and harmonized procedures across all sites, we aimed to eliminate this methodological noise and facilitate the detection of genuine spatial patterns in MP pollution. This design allowed us to evaluate spatial patterns in MP contamination across two ecological scales: among lakes and among countries. We focused on a set of widely recognized drivers of MP variability in freshwater ecosystems, including nutrient concentrations (total nitrogen, total phosphorus, and chlorides), shoreline urbanization intensity (as quantified by the shoreline urbanization index, SUI), seasonal differences, lake typology (reference vs. eutrophic), and proximity to potential point-source pollution. The SUI provides a standardized measure of anthropogenic pressure on lake shorelines, calculated as the ratio of shoreline with visible human development (e.g., artificial surfaces, roads, built-up structures) to total shoreline length. This index reflects shoreline transformation due to land use and infrastructure and has previously been shown to correlate with environmental pollution and habitat alteration (Pol et al. 2023). These variables represent both localised environmental conditions and broader socio-environmental pressures.

By removing methodological noise, we expected to uncover clearer and more consistent patterns in MP abundance and characteristics, namely, size distribution, shape, polymer composition, and colour. Specifically, we aimed to determine whether these patterns reflect ecological gradients, shoreline transformation as quantified by the shoreline urbanization index (SUI), or national-scale differences in environmental management. This approach provides a more reliable basis for understanding the true environmental determinants of freshwater MP pollution and for informing future monitoring efforts in diverse geographic settings.

## Materials and methods

### Study site

**Fig. 1:**
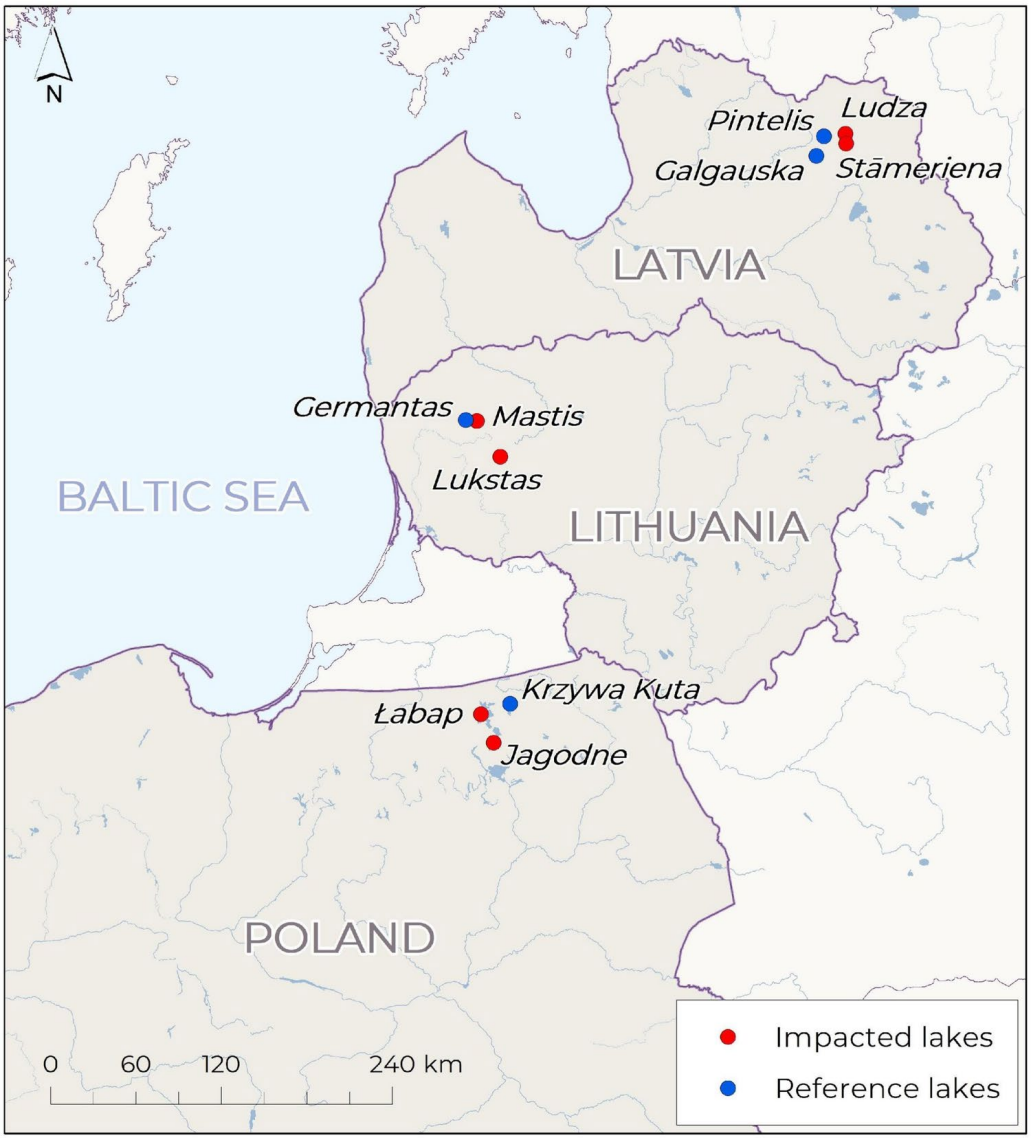
Map showing the locations of the examined lakes

The study was conducted in ten lakes across three Baltic Sea countries: Poland, Lithuania, and Latvia (see Supplementary Table S1 for characteristics of studied lakes). The selection included both lakes of significant importance for tourism and reference lakes with minimal or no tourist activity (Fig. 1). Tourist-oriented lakes were chosen based on their well-established recreational uses, such as swimming, kayaking, and fishing, as well as the presence of nearby hotels and frequent occurrence of outdoor events, ensuring they attracted a substantial number of visitors. In Poland, Lake Jagodne (often used as a flow-through lake to access other lakes in the Great Masurian Lakes by boat) and Lake Łabap (frequently used for sailing) were selected due to their popularity and extensive tourism infrastructure in the Masurian region (northeastern Poland). In Lithuania, the study focused on Lake Mastis, located in the town of Telšiai, and Lake Lukstas, both recognized for their recreational appeal (northeastern Lithuania). In Latvia, Lake Ludza and Lake Stāmeriene, situated in the eastern part of the country, were included due to their active tourism-related activities. Reference lakes, selected to serve as baselines for assessing the impact of tourism on MP pollution, included Lake Krzywa Kuta (Poland), Lake Germantas (Lithuania), and Lake Pintelis and Lake Galgauskas (Latvia). This selection allowed for a comparative analysis of the interactions between tourism activities and environmental sustainability across the studied lakes.

### Lake sampling

All selected lakes were sampled for surface MP contaminants and water chemistry (total phosphorus, total nitrogen, and chloride) during three key periods in 2023: spring (May), summer (August), and autumn (October), representing conditions before, during, and after the peak tourism season. Sediment MP pollution was sampled once in 2023 to provide a snapshot of accumulated MP deposition over the year.

Both water and sediment samples were collected by the same research team, following a harmonized protocol, and using identical equipment across all sites and countries. This ensured that all samples were collected using identical Manta nets (300 µm mesh size) and processed with the same FTIR-based polymer identification workflow, under identical laboratory conditions, including procedural blanks and positive controls to ensure analytical consistency. Such full standardization minimized procedural variability and maximized comparability between sites, directly addressing limitations of earlier multi-lake studies.

In each lake, three surface water samples (replicates) and sediment cores were collected. Sediment samples covered ecologically distinct and functionally relevant zones: (1) the deepest central basin, considered the primary sediment accumulation area in limnology and paleolimnology; (2) a representative coastal zone; and (3) a site adjacent to a potential point source of pollution (e.g., hotel pier, marina, or drainage outlet). This spatially stratified design was applied consistently across all lakes.

#### Surface water MP sampling

Surface water samples were taken using a manta net (Hydro-Bios, mesh 300 µm) towed from the stern for 20 min along a long transect within each zone (one tow per zone per season). The filtered volume per tow ranged from 0.74 to 36.3 m^3^ (mean 14.61 m^3^), recorded with a mechanical flow meter (Hydro-Bios). After each tow, the net was rinsed externally to concentrate material in the cod end, which was removed over a metal tray, inverted, and rinsed with filtered water. Each sample was transferred into a pre-cleaned glass tray, covered with aluminium foil and a metal lid, and stored at 2–4 °C until processing.

#### Sediment MP sampling

Sediment cores were collected from different zones of the lake using a kayak-mounted sediment corer (KC Denmark, model 13.030) with an acrylic tube (52-mm internal diameter). In some cases (e.g., Lake Jagodne), logistical constraints required sampling from deep areas close to, but not exactly at, the maximum depth. For MP analysis, the top 5 cm of each core was extracted. Samples were transferred into pre-cleaned glass vials, sealed with aluminium foil and metal lids, and stored at 2–4 °C until further processing.

#### Water sampling for chemical analysis

Water for chemistry was collected at 0.5 m below the surface above the deepest point of each lake using a sterile, autoclaved 1-L glass bottle (one bottle per season). Bottles were submerged carefully to avoid contamination and to preserve in situ composition prior to analysis.

### Sample processing and analysis

#### Surface water and sediment MP samples

All MP samples were processed at the Microplastic Laboratory of the Latvian Institute of Aquatic Ecology following established protocols (Barone et al. 2024). Preparatory steps (see Supplementary Table S2 for treatment applied to surface water and sediment MP samples) included oxidation, surfactant application, enzymatic digestion (cellulase and viscozyme in acetate buffer at pH 4.8, alkalase and protease in TRIS buffer at pH 8.2), and density separation with sodium polytungstate (density 1.75 g × cm^3^). Samples were filtered through a 200-µm stainless steel sieve (RETSCH) during purification and finally collected on glass fibre filters with a pore size of 1.2 µm.

Potential plastic particles (≥ 300 µm) were visually identified, sorted, and counted using a Leica DM400 B LED light microscope with a DFC 295 camera and Leica Application Suite V4.1 software. Particles were categorised by shape (fibres or fragments), colour, and size. Large particles were analysed using attenuated total reflection-Fourier transform infrared (ATR-FTIR) spectroscopy (Thermo Scientific, Nicolet iS20). For smaller particles that could not be picked manually, a hot needle test (Postma 2022) was performed on up to ten particles per sample filter to confirm their synthetic nature.

#### Quality control measures

Quality control procedures followed Dimante-Deimantovica et al. (2022). Samples were handled in fume or laminar flow hoods, using only glass, metal, or PTFE equipment, thoroughly rinsed with filtered Milli-Q water before use. Samples were covered with aluminium foil when not being processed or when placed in a shaking heating bath. Laboratory personnel wore cotton lab coats and nitrile gloves throughout all procedures.

Negative procedural controls (blanks) for both field and laboratory steps were processed in parallel with each series of environmental samples to assess potential contamination. In surface-water laboratory blanks, the average was ~ 3 particles per batch; field blanks showed similar averages, representing 0–71% of paired sample counts. For sediments, laboratory blanks averaged ~ 3.5 particles, while field blanks averaged ~ 7.6 particles (7.4–111.1% of paired sample counts). High blank/sample ratios occurred only in a few isolated cases. The content of MP particles in field and laboratory blanks was reported but not used to adjust the final results. The decision not to apply a numerical blank correction (e.g., via minimum detectable amount) was intentional, as there is currently no widely accepted or standardized method for blank contamination adjustment in MP research (Munno et al. 2023).

Positive controls (spikes) were conducted in triplicate by adding 100 standardized red PS beads (diameter = 100 µm, density 1.05 g cm⁻^3^; Sigma-Aldrich, product number 56969-10ML-F) to both water and sediment samples. The spiked samples underwent the same treatment as field samples, except that a 50-µm sieve was used instead of a 200-µm sieve. Recovery rates were 84–94% for sediments and 90–95% for surface-water samples.

#### Water chemicals’ samples

Total phosphorus (*P*_tot_) in lake water samples was determined using inductively coupled plasma mass spectrometry (ICP-MS) with a NexION 300D ICP mass spectrometer (PerkinElmer SCIEX, USA). Total nitrogen (*N*_tot_) concentrations were measured using the Merck Spectroquant® Nitrogen Total Cell Test (0.5–15.0 mg × L^−1^) (cat. no. 1.14537.0001) according to the manufacturer’s instructions. *N*_tot_ was defined as the sum of total Kjeldahl nitrogen (organic and reduced nitrogen, including ammonia), nitrate, and nitrite. Prior to analysis, samples were thermally digested at 129 °C using a Prestige Medical tabletop autoclave to convert organic and inorganic nitrogen compounds to nitrate, which then reacted to form a photometrically detectable compound. Chloride (Cl⁻) concentrations (0.5–400 mg × L^−1^), used as an indicator of anthropogenic pressure, were determined using Merck’s Spectroquant® Chloride Test reagents (cat. no. 1148970001) following the manufacturer’s instructions. The absorbance of the coloured reaction product in the chloride assay was measured with a Shimadzu UV-1201 V spectrophotometer.

### Shoreline urbanization index

Hydrographic data for the lakes were obtained from the Atlas of the Lakes of Poland (Jańczak 1999). Where data were unavailable, analysis was conducted using current land use maps, particularly the CORINE Land Cover (CLC 2018) dataset provided by the Polish Chief Inspectorate for Environmental Protection (GIOŚ) and the Map of the Hydrographic Division of Poland (MPHP10k). This dataset was developed by the National Research Institute of Meteorology and Water Management (IMGW-PIB) in cooperation with the National Water Authority (KZGW), the Main Office of Geodesy and Cartography (GUGiK), the Government Centre for Security, and the National Institute of Telecommunications–State Research Institute. Data on catchment areas and wastewater treatment plant (WWTP) capacities were obtained from CORINE Land Cover (CLC 2018).

To ensure objective results, a simple SUI was applied to represent the proportion of anthropogenically modified land along the entire lake shoreline (Pol et al. 2023). The index was calculated using the formula: SUI = SLu × SL^−1^ × 100%, where SLu is the urbanised shoreline length (m) and SL is the lake shoreline length (m). For lakes with insufficiently detailed maps, the urbanized shoreline length was further adjusted based on comprehensive field surveys.

### Data analysis

To assess the influence of the SUI and concentrations of Cl⁻, *N*_tot_, and *P*_tot_ on MP abundance in surface waters, redundancy analysis (RDA) with stepwise forward selection was applied to log-transformed data to meet model assumptions. Statistically significant predictors were identified using permutation-based significance testing. For variables selected through RDA, either a generalised additive model (GAM) was fitted to explore potential nonlinear relationships, or a generalised linear model (GLM) was used to test for linear associations between environmental variables (SUI, Cl⁻, *N*_tot_, *P*_tot_) and MP concentrations in both surface water and sediment samples.

Depending on the structure of the dataset, different statistical tests were used to assess differences in MP metrics, namely, concentration, shape, size, polymer type, and colour (hereafter, M_Pconc_, MP_shape_, MP_size_, MP_species_, and M_Pcolor_), across countries, individual lakes, lake types (L_types_: reference lakes with no tourism pressure vs. study lakes with tourism impact), seasons, and within-lake sampling locations (Spoint: near vs. away from point-source pollution). These included the Mann–Whitney *U* test (applied exclusively to lake type comparisons), the Kruskal–Wallis rank-sum test with Dunn’s post hoc procedure and Bonferroni correction, and the approximate K-sample Fisher-Pitman permutation test, supplemented by pairwise comparisons using either exact or asymptotic methods with false discovery rate (FDR) correction.

Seasonal comparisons were conducted only for surface water samples, while lake type comparisons were applied only to sediment samples. Analyses were performed on both absolute values and proportional data, with the exception of MP_conc_ and MP_species_ (analysed only as absolute counts) and MP_color_ (analysed only as proportions). All statistical analyses and data visualizations were carried out in RStudio (version 2024.12.1–563) using the packages *vegan*, *mgcv*, *coin*, *dunn.test*, *permute*, and *ggplot2*.

## Results

### General characteristics of MPs across all lakes

**Fig. 2:**
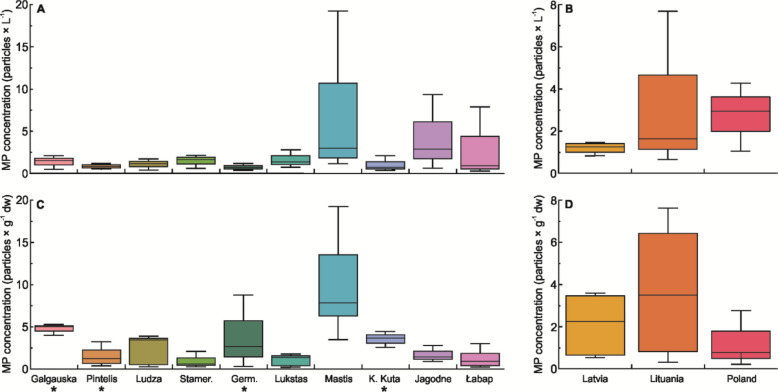
MP concentrations in water (**A, B**) and sediment (**C, D**) samples. Panels **A** and **C** show concentrations for individual lakes, while panels **B** and **D** present values pooled by country. No differences between lakes or countries were statistically significant. * indicates reference lakes

**Fig. 3:**
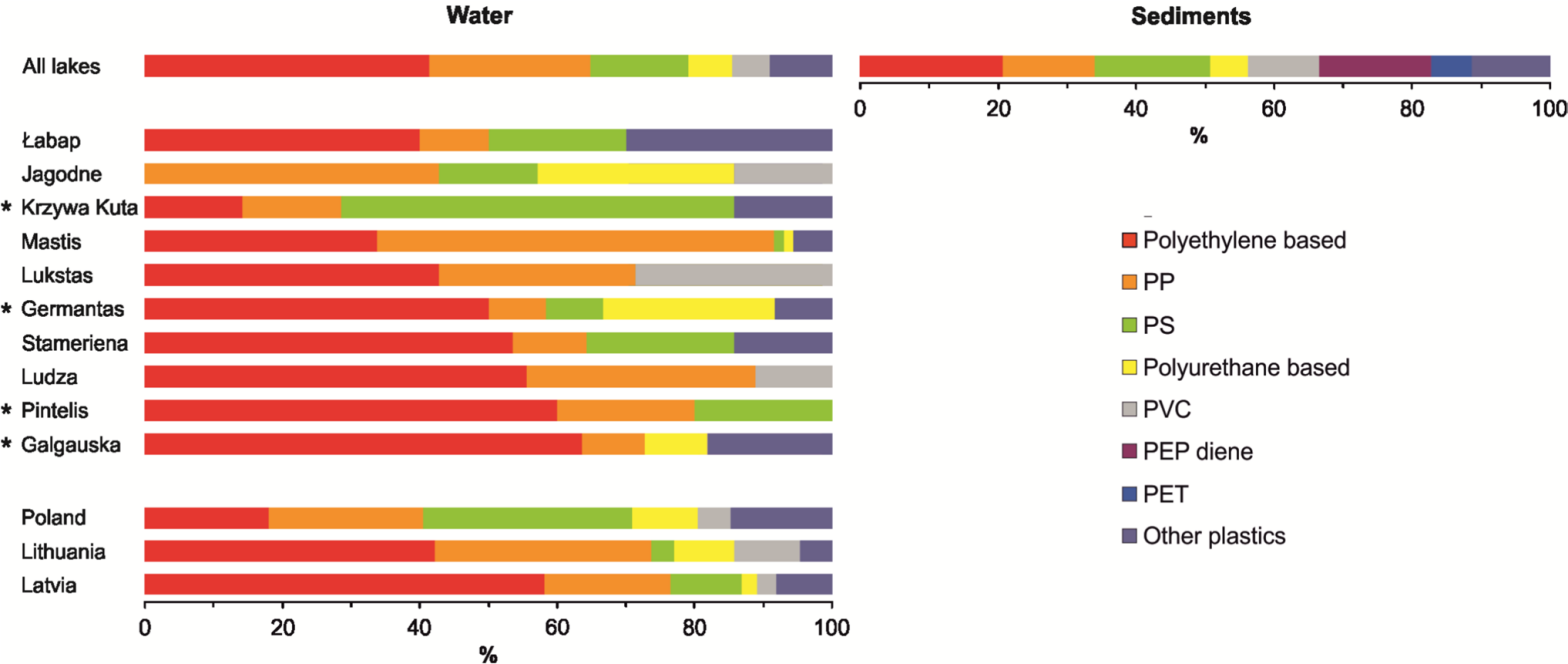
The proportion of different types of MPs in surface water samples, shown separately for each lake, grouped by country, and for all lakes combined. Additionally, the figure presents the proportion of different MP types in sediments across all lakes. * indicates reference lakes

**Fig. 4:**
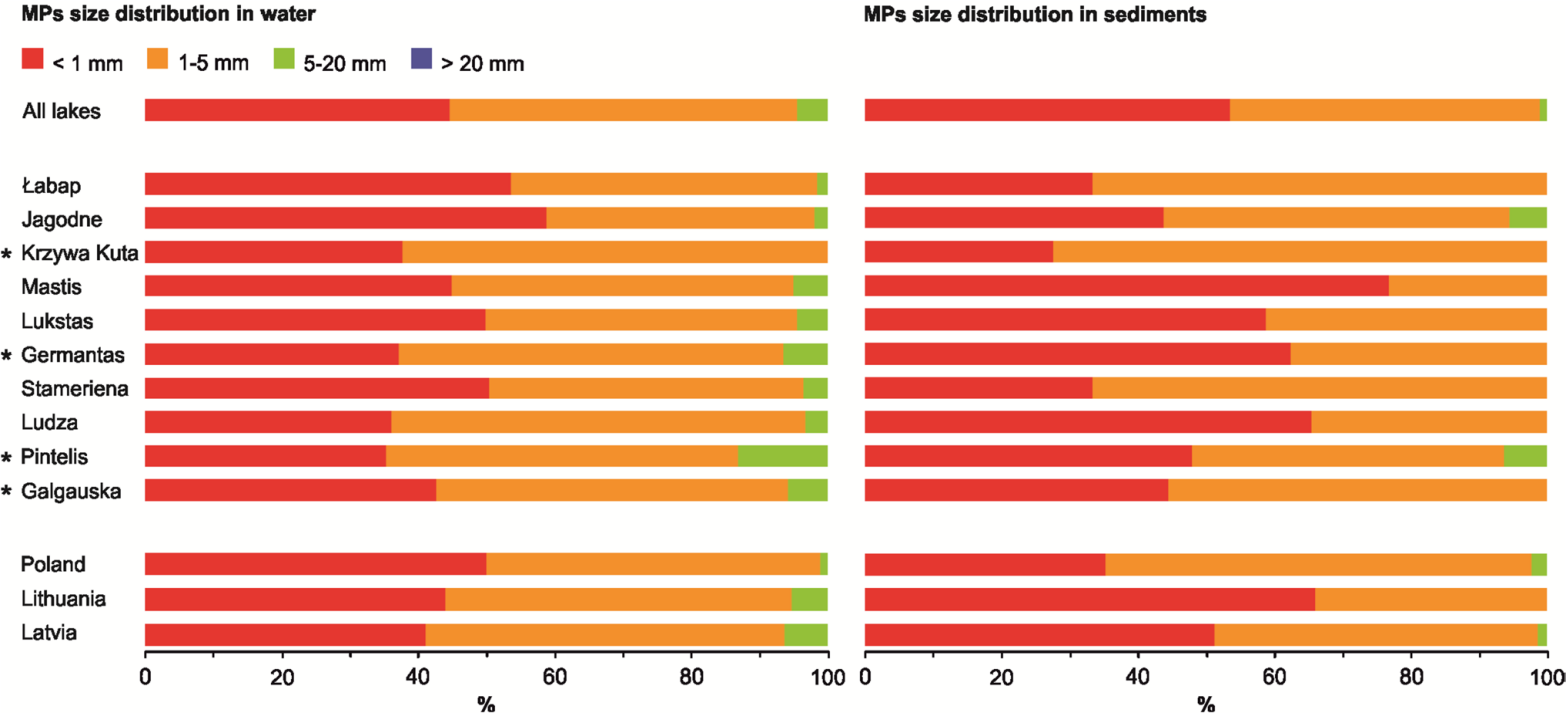
Proportional distribution of MP size classes in surface water and sediment samples in all lakes combined, in individual lakes, and in lakes grouped by country. * indicates reference lakes

**Fig. 5:**
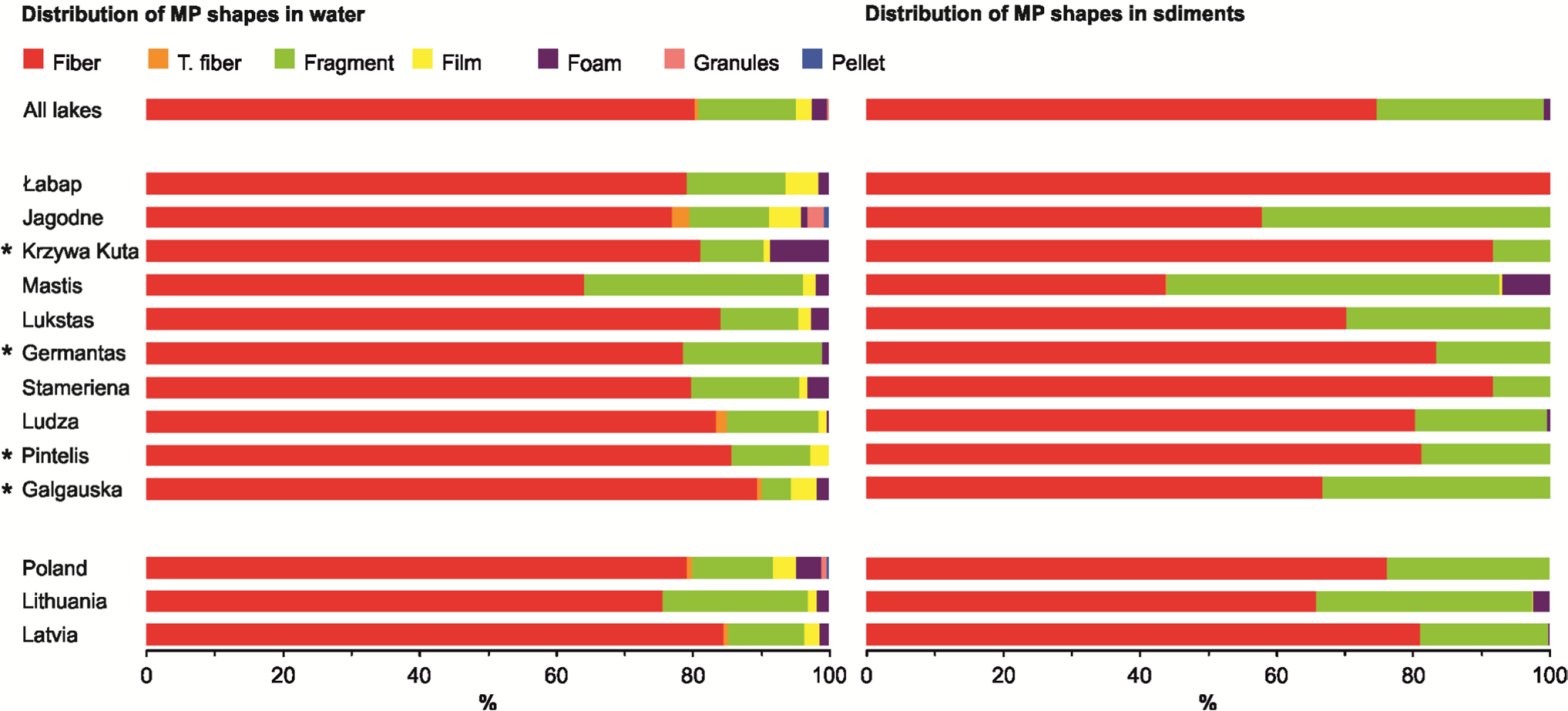
Proportional distribution of MP shapes in surface water and sediment samples in all lakes combined, in individual lakes, and in lakes grouped by country. * indicates reference lakes

**Fig. 6:**
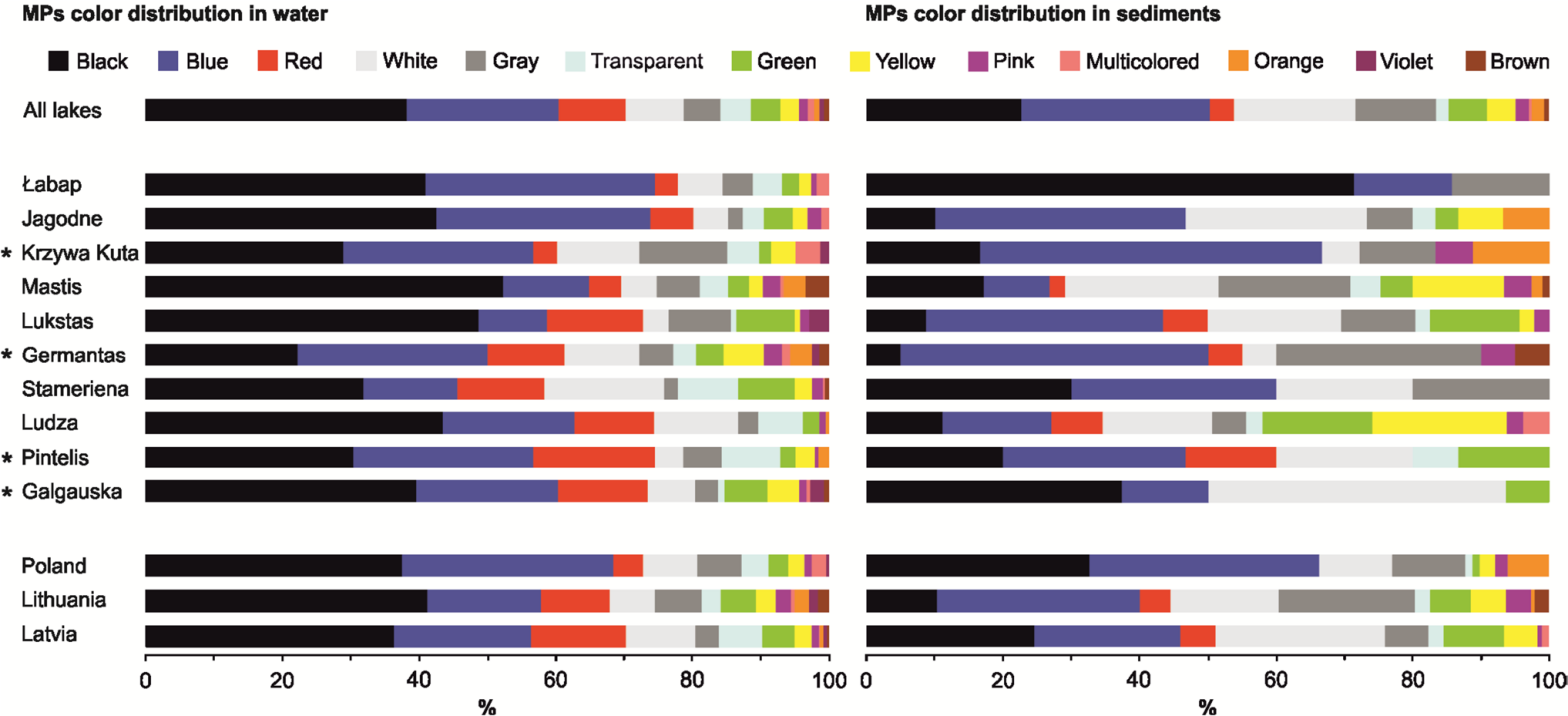
Proportional distribution of MP colours in surface water and sediment samples in all lakes combined, in individual lakes, and in lakes grouped by country. * indicates reference lakes

The mean MP concentrations in surface waters ranged from 0.67 particles L⁻^1^ in Lake Germantas (0.50 and 0.80 particles L⁻^1^ in summer and autumn, respectively) to 7.68 particles L⁻^1^ in Lake Mastis (19.3 and 1.58 particles L⁻^1^ in summer and spring, respectively). In sediments, concentrations ranged from 0.09 particles kg⁻^1^ DW in Lake Łabap (0.08 and 0.13 particles kg⁻^1^ DW in autumn and summer in coastal waters, respectively) to 3.90 particles kg⁻^1^ DW in Lake Mastis (2.2 particles kg⁻^1^ DW in summer in the deep part of the lake and 6.7 particles kg⁻^1^ DW in summer near the pollution source, respectively) (Fig. 2). No statistical differences were detected between individual lakes or between lakes pooled by country, for either water or sediment samples (Fig. 1). Across all lakes, PE (polyethylene) and PP (polypropylene) were the most commonly identified polymer types in surface waters, followed by PE-acrylic acid and PS (polystyrene). In sediments, the distribution was similar, though with relatively higher proportions of PS, PVC (polyvinyl chloride), and PEP diene (polyethylene-propylene-diene elastomer) (Fig. 3). Particles smaller than 1 mm and those between 1 and 5 mm dominated both compartments (Fig. 4). Fibres were the predominant shape in both water and sediment samples, followed by fragments (Fig. 5). The most frequently observed colours were black, blue, and white. Grey particles were more abundant in sediments, while red particles appeared more commonly in surface waters (Fig. 6).

### Relationships and differences among lakes

Among the environmental and anthropogenic variables tested, the SUI was the only significant predictor of MP abundance in surface waters (RDA; *F* = 10.876, *p* = 0.02). A nonlinear relationship was confirmed (GAM; *F* = 12.94, *p* = 0.0087), with SUI explaining 73.7% of the variance (adjusted *R*^2^ = 0.737; Fig. S1). When country was added to the model, it did not improve the prediction (GAM; *p* > 0.05), indicating that variation among countries was negligible once SUI was accounted for. SUI remained a significant predictor (GAM; *F* = 9.083, *p* = 0.0291). No significant associations were found between MP abundance and nutrient concentrations (Cl⁻, *N*_tot_, and *P*_tot_), nor with proximity to point-source pollution, lake type, or season (see Supplementary Table S3 for an overview of statistical testing results based on individual factor analysis). However, seasonal variation affected MP composition: fibre particles were less abundant in spring than in summer (permutation test; *p* = 0.0241), while foam particles were more abundant in spring compared to autumn (permutation test; *p* < 0.001) (see Supplementary Table S3 for an overview of statistical testing results based on individual factor analysis). No significant differences were observed in MP concentration, size, shape, polymer type, or colour among individual lakes (Kruskal–Wallis, Wilcoxon, and permutation tests; Figs. 2, 3, 4, 5, and 6, Table S3; overview of statistical testing results based on individual factor analysis). These findings support the conclusion that, among lakes, spatial variation in MP contamination is relatively limited when methodology is standardized.

### Regional differences among countries

When country was added as a predictor of MP abundance, no significant effect was found (GAM; *p* > 0.05) (see Supplementary Table S3 for an overview of statistical testing results based on individual factor analysis), confirming that MP concentrations do not systematically differ between countries when shoreline urbanization is accounted for. However, several MP characteristics showed differences among countries (see Supplementary Table S3 for an overview of statistical testing results based on individual factor analysis). In surface waters, Latvian and Lithuanian lakes had significantly higher proportions of 5–20 mm particles than Polish lakes (Kruskal–Wallis; *p* = 0.016; Dunn’s test: Latvian vs. Polish *p* = 0.0203; Lithuanian vs. Polish *p* = 0.016). In sediments, Lithuanian lakes had a higher share of < 1 mm particles and a lower share of 1–5 mm particles compared to Polish lakes (Kruskal–Wallis; *p* < 0.05; Dunn’s test: *p* = 0.012 and *p* = 0.016, respectively).

Differences in MP colour composition in surface waters were also observed. Statistically significant variation was found in the proportions of blue, brown, and multicoloured particles (permutation test; all *p* < 0.05) (see Supplementary Table S3 for an overview of statistical testing results based on individual factor analysis), although post hoc tests did not identify consistent pairwise differences. Similarly, PP abundance in surface waters varied significantly by country (permutation; *p* = 0.0416), but pairwise comparisons were inconclusive.

The only MP characteristic that differed between lake types was the absolute abundance of < 1 mm particles in surface waters, with study lakes showing higher counts than reference lakes (Mann–Whitney test; *p* = 0.0309) (see Supplementary Table S3 for an overview of statistical testing results based on individual factor analysis). In sediments, a significant difference in the proportional distribution of < 1 mm particles was observed between sampling points (Kruskal–Wallis test; *p* = 0.0235) (see Supplementary Table S3 for an overview of statistical testing results based on individual factor analysis), but post hoc tests did not resolve the specific sources of this variation.

## Discussion

### General insights and methodological context

Lakes function both as sinks and transfer zones for MP pollution due to their restricted water exchange and proximity to multiple anthropogenic sources (Dusaucy et al. 2021; Tockner 2021). In these systems, the environmental fate of MPs is shaped by synergistic physicochemical processes such as fragmentation, sedimentation, aggregation, and resuspension (Besseling et al. 2017; Guo et al. 2024) in combination with biological interactions (i.e., ingestion, gut passage, faecal packaging, and biofilm colonization). These processes accelerate particle breakdown and modify transport pathways (Babkiewicz et al. 2025; Pukos et al. 2023). Understanding these combined mechanisms is critical for predicting spatial patterns and assessing ecological risks under realistic environmental conditions (Besseling et al. 2017; Guo et al. 2024).

Yet, the ability to detect such patterns and identify ecological drivers has long been constrained by methodological inconsistencies (Chen et al. 2024a; Setälä et al. 2016; Martí et al. 2020). Differences in sampling gear, mesh size, polymer identification, and quality-control protocols are known to increase variability and mask environmental patterns. As highlighted by Earn et al. (2021) in their review of Laurentian Great Lakes studies, such methodological disparities can limit comparability even within a single region, underscoring the value of harmonized protocols for detecting true environmental signals.

In our study, all stages from field collection to laboratory analysis were harmonized: identical manta nets, mesh sizes, and sample volumes were used, with FTIR-based polymer identification and consistent QA/QC procedures (procedural blanks, positive controls). Field and laboratory work was conducted by the same team, eliminating operator-based variability. While harmonized protocols have previously been used in single-country or single-catchment studies, our survey represents the first fully standardized, multi-country lake MP assessment applied across the Baltic Sea region. This design removes methodological noise and enables more reliable detection of subtle but ecologically relevant differences.

This unified approach allowed us to examine MP patterns at two complementary scales: between individual lakes differing in morphometry and shoreline use and between countries with contrasting population density, wastewater infrastructure, and environmental governance. It also provides a robust baseline for future MP monitoring, where eliminating methodological artefacts will be crucial for detecting fine-scale environmental signals.

### MP characteristics across all lakes

In surface water, the average MP concentration was 2.30 particles × L⁻^1^ (median 1.42; CV 69.13%). These values were moderately higher than the European lake mean reported by Jachimowicz et al. (2026) of 0.35 ± 0.95 particles × L⁻^1^ (median ~ 0.05–0.10), but variability in that dataset was almost four times higher (CV 271.43%). In sediments, our mean concentration was 1.08 particles × g⁻^1^ DW (median 0.3960; CV 107.5%), close to the European average of 1.69 ± 4.23 particles × g⁻^1^ DW (median ~ 0.2–0.4), yet again with more than double the variability (CV 250.3%). These contrasts show that while mean concentrations in our dataset are within the same order of magnitude as broader European surveys, variability is considerably lower, especially for surface waters, which can at least in part be attributed to reduced methodological noise due to harmonized sampling and analytical methods.

Lake Jagodne, sampled both here and by Pol et al. (2023), showed 5.8 times higher MP concentrations in our dataset. This difference likely reflects the variability among chosen sampling methods (6 ₓ 5 L water sampling versus Manta trawling), MP isolation methods and analysed size fraction. 

Polymer composition was dominated in both compartments by PE and PP, with PE–acrylic acid and PS also prevalent in surface waters. In sediments, denser polymers such as PS and PVC, as well as PEP diene, were proportionally more abundant, likely reflecting greater sedimentation potential; biofilm growth or aggregation could also enhance settling rates irrespective of density (Amaral-Zettler et al. 2021; Pukos et al. 2023).

Particles < 1 mm and those 1–5 mm comprised the majority of MPs in both compartments. Although the 300 μm mesh excluded the smallest size fraction from capture, FTIR identification was still possible for some sub-300 μm particles retained within aggregates or on fibres. Fibres dominated overall, particularly in sediments, consistent with major sources most likely such as textiles, fishing lines, and ropes, and with their high propensity for biofilm colonization and deposition.

Black, blue, and white were the most common colours in both compartments, with grey more frequent in sediments and red more frequent in surface waters. Colour composition may be influenced by source material and post-depositional processes such as photodegradation and biofilm growth.

The reduced variability in this harmonized dataset, representing the first such cross-country comparison for lakes in three countries, provides a strong basis for detecting environmental drivers and suggests that many previously reported between-region differences may partly arise from methodological artefacts rather than existing ecological variation.

### MP characteristics among lakes

Applying a unified sampling protocol revealed relatively fine spatial patterns across the ten lakes. While no major differences were detected in overall MP abundance or basic characteristics, several ecologically meaningful relationships emerged.

The SUI was the most consistent environmental predictor, showing a positive association with MP concentrations in surface waters. This supports growing evidence that shoreline infrastructure and human activities, particularly tourism, are stronger drivers of MP input into lakes than broader catchment-level variables (Pol et al. 2023). The absence of a similar relationship in sediments suggests that MPs in the water column reflect more recent or episodic inputs, whereas sediments integrate longer-term deposition patterns shaped by lake-specific hydrodynamics (van Emmerik et al. 2022).

In sediments, higher SUI values were linked to a greater share of fragments and films and a lower proportion of fibres, likely reflecting differences in the origin and transport behaviour of MP types in urban run-off. Similarly, polymer composition in sediments revealed associations with shoreline development: higher proportions of PET, PS, and PMMA were observed in lakes with elevated SUI values. These results align with earlier reports that urban run-off delivers distinctive polymer and shape profiles (Jachimowicz et al. 2026; Zhao et al. 2024; Chen et al. 2024a).

No consistent associations emerged between MP concentrations and chemical water quality parameters such as total nitrogen, total phosphorus, or chlorides. While MPs and nutrients may originate from overlapping anthropogenic sources, their environmental fates are governed by distinct processes: nutrients, being primarily dissolved or associated with fine particulates, are subject to rapid biological cycling and solute transport, whereas MPs are transported as discrete particles influenced by size, density, and morphology (Ross et al. 2023; Cho et al. 2023). This decoupling suggests that monitoring programmes should treat MPs and nutrients as complementary but separate indicators of anthropogenic impact.

Trophic state, previously linked to MP levels in review studies (Liu et al. 2022; Jachimowicz et al. 2026), did not predict MP abundance in our dataset and showed no correlation with polymer type, shape, or colour. The only exception was a slightly higher share of particles < 1 mm in eutrophic lakes, consistent with global observations (Liu et al. 2022), possibly resulting from enhanced fragmentation of plastics in more productive systems where elevated biological activity contributes to breakdown of larger particles (Babkiewicz et al. 2025).

Within-lake spatial heterogeneity was limited: sites near putative pollution sources did not consistently show higher sediment MP concentrations. This may reflect hydrodynamic mixing that dilutes local inputs, or MP inputs that are diffuse and episodic rather than persistent. Although our sampling design included three distinct cores per lake, we did not replicate cores within the same functional zone, which could have captured small-scale heterogeneity in MP deposition. We acknowledge this limitation and suggest that future studies incorporate within-sampling site replication to better quantify fine-scale variability. Our aim was to capture major depositional contexts with a pragmatic, ecologically grounded protocol, not to resolve fine-scale within-lake variability.

These findings contrast with observations from more densely urbanized or industrial systems, where pronounced spatial gradients in MP abundance have been documented (Dris et al. 2018; Jachimowicz et al. 2026). However, the relatively small number of lakes and limited replication of source-related sampling points in our study may have reduced statistical power to detect such patterns.

Seasonal patterns in total MP concentrations were weak, but compositional shifts were evident: fibres were less common in spring, while foams were more prevalent, likely reflecting seasonal differences in stratification, run-off, and recreational activity. Such temporal signals are rarely captured in MP studies, which often rely on single-season data (Jachimowicz et al. 2026). By incorporating multiple seasons, our study provides a more comprehensive and temporally representative picture of MP dynamics in freshwater systems.

Overall, the modest variation in MP concentrations among lakes, despite differences in morphometry, shoreline use, and human activity, demonstrates that standardized methodology reduces apparent heterogeneity and helps identify key drivers such as shoreline development. This supports broader adoption of harmonized frameworks for detecting subtle but ecologically relevant patterns in MP distribution. As these lake-level findings suggest, standardization also sets the stage for more robust analyses across broader geographic boundaries, which is explored in the next section.

### MP characteristics in lakes across national contexts

Despite contrasts in population density, waste management systems, and shoreline development, no substantial differences in overall MP loads were observed between the three countries. Poland has a population density more than three times higher than Latvia and nearly triple that of Lithuania, alongside the highest GDP per capita and the most industrialised economy, with strong heavy manufacturing sectors (Table 1). Wastewater treatment coverage is extensive, though some older systems remain in operation. In contrast, Latvia and Lithuania have lower population pressures and more modest industrial activity, with economies partly based on agriculture, forestry, and low‐ to medium‐tech manufacturing (Table 1). Wastewater treatment is advanced in urban centres but remains incomplete in rural areas, and seasonal tourism can add episodic plastic inputs. All three countries operate under EU‐level plastic pollution regulations, but enforcement stringency and public awareness vary (Table 1). These differences could, in principle, influence baseline MP inputs to lakes; nonetheless, in this particular study, we did not observe substantial variation in measured MP concentrations.

Mean concentrations reached 1.3 ± 0.4, 3.4 ± 1.2, and 2.8 ± 0.9 particles L⁻^1^ in surface waters and 0.9 ± 0.3, 1.8 ± 0.7, and 0.7 ± 0.2 particles g⁻^1^ DW in sediments for Latvia, Lithuania, and Poland, respectively. These values confirm that, when sampling and analytical procedures are fully standardized, apparent geographic differences in MP abundance largely diminish. Comparable benefits of harmonization have been demonstrated elsewhere: for instance, in the Laurentian Great Lakes, adopting unified sampling meshes, extraction solutions, and FTIR workflows across multiple monitoring campaigns substantially reduced inter-study variability and enabled detection of consistent cross‐lake patterns that were previously obscured by methodological noise (Earn et al. 2021). Similarly, the EU Horizon 2020 BASEMAN project showed that coordinated protocols for MP monitoring across European marine and freshwater systems allowed for robust, cross‐regional comparisons and long‐term pollution trend assessments (Frias et al. 2018).

Particular regional differences in composition were detected. Latvian and Lithuanian lakes had a higher proportion of larger MPs (5–20 mm) in surface waters and greater colour diversity, particularly blue, brown, and multicoloured particles. Lithuanian lake sediments contained more MPs < 1 mm, suggesting country-specific influences on degradation, transport, or retention. Polymer composition varied slightly, with surface-water PP abundance differing among countries, possibly reflecting differences in plastic use or wastewater treatment efficiency in removing specific polymer types. These composition-based differences complement the lake-level patterns reported in ‘MP characteristics among lakes’, where shoreline urbanization influenced polymer and shape profiles in sediments.

Wastewater treatment infrastructure offers a plausible explanation for some of these patterns. In Poland, more extensive and modernized facilities may better retain smaller, fibre‐rich MPs, whereas outdated or rural plants in parts of Latvia and Lithuania may be less effective at removing larger or more heterogeneous particles, enabling their release via municipal or hospital effluents (Pashaei et al. 2023). Recreational use may also play a role: in Latvia and Lithuania, seasonal tourism and shoreline activities could add episodic inputs of plastics with distinct morphologies and colours, whereas the Polish lakes sampled here were in more industrialised or densely populated areas, where littering and stormwater discharge may carry MPs differently.

Globally, large differences in MP concentrations (e.g., Asia vs. Europe) are often attributed to higher urbanization pressure, unappropriated waste management, and, at least in part, to methodological variation (Jachimowicz et al. 2026), including disparities in flotation solutions, particle extraction efficiency, and the detection limits of spectroscopic methods such as FTIR and Raman (Yin et al. 2023). These factors contribute to inconsistent recovery rates and complicate interpretation; when protocols are harmonized, intra‐continental differences tend to be smaller, and while common polymer types (PE, PP) and shapes (fibres, fragments) often dominate across regions, composition metrics can still reveal localised influences of infrastructure, land use, and environmental conditions.

In summary, cross-country differences in MP concentrations were minimal, while composition-level signals were linked to human activity, infrastructure, and environmental conditions. The ability to resolve these patterns reflects the reduction of methodological noise achieved through harmonization. Our results provide a strong regional-scale example that coordinated protocols can minimize analytical variability, allowing composition-level contrasts to emerge. Building on the evidence provided by our study, future research should also prioritize the development and consistent application of unified sampling and analytical protocols, ensuring that methodological artifacts are minimized and that spatial patterns in MP pollution are accurately resolved.

## Conclusions

Applying identical sampling gear, mesh size, QA/QC procedures, and FTIR‐based polymer identification across ten lakes in Latvia, Lithuania, and Poland removed methodological noise that used to hide true environmental patterns. This harmonization revealed shoreline urbanization as the dominant predictor of surface‐water MP concentrations, while chemical parameters such as nutrients and chlorides showed no consistent effect. Despite contrasts in population density, wastewater infrastructure, and governance, MP concentrations were remarkably similar among countries. Only MP variations in size, colour, and selected polymer types were detected. These patterns suggest that many reported regional contrasts in previous studies may be methodological artefacts rather than genuine environmental signals. Our findings highlight that urban shoreline development is a critical factor driving MP accumulation, underscoring the importance of considering urbanization in future environmental assessments. Together with methodological considerations outlined by Yin et al. (2023), our results demonstrate that coordinated approaches can reduce analytical variability and uncover fine‐scale composition‐level patterns. By showing that protocol harmonization enables detection of such patterns, this work sets a precedent for future large‐scale MP assessments. Applying unified methodologies across broader ecological and socioeconomic gradients and integrating seasonal and long‐term monitoring will generate the robust evidence base needed for effective plastic pollution management in inland waters. Future standardized surveys should also address research gaps identified by Yin et al. (2023), including the need for long‐term toxicity assessments in lacustrine environments, investigation of MP degradation and mineralization in organic‐rich waters, and evaluation of how climatic and geological lake histories influence MP fate and impacts.

## Supplementary information

Below is the link to the electronic supplementary material.

ESM 1 Additional data on lake characteristics, MP sample processing methods, statistical testing, and GAM model outputs are provided in the supplementary materials. (DOC 126 KB)

## Data Availability

The authors declare that the data supporting the findings of this study are available within the paper and its Supplementary materials.
