# Supplementary material for: Microplastics in the Baltic Sea region lakes—standardized insights reveal urban shoreline as key driver
Source: Environ Sci Pollut Res Int. 2025 Nov 21;32(47):27052–67. doi: 10.1007/s11356-025-37103-x (PMC12675769; doi:10.1007/s11356-025-37103-x)
Supplement: Supplementary file 1 — Additional data on lake characteristics, MP sample processing methods, statistical testing, and GAM model outputs are provided in the supplementary materials. (DOC 126 KB) [file 11356_2025_37103_MOESM1_ESM.doc]

**Supporting information**

**Microplastics in the Baltic Sea region lakes – standardized insights reveal urban shoreline as key driver**

**Ewa Babkiewicz^a,b*^, Elina Vecmane^c^, Magdalena Fuk^d^, Magdalena Jurgielewicz^d^, Agnieszka Koniuk^d^, Eliza Kurek^a^, Piotr Maszczyk^b^, Magdalena Michalska-Kacymirow^a^, Daiva Jonuskiene^e^, Jolanta Norvaišienë^e^, Valentina Burdukovska^c^, Inta Dimante-Deimantovica^f^, Juris Tunēns^c^, Wojciech Pol^g^, Ewa Bulska^a^**

^a^ University of Warsaw, Biological and Chemical Research Centre, Warsaw, Poland

^b^ University of Warsaw, Faculty of Biology, Department of Hydrobiology, Warsaw, Poland

^c^ Latvian Institute of Aquatic Ecology, Riga, Latvia

^d^ Foundation for the Protection of Great Masurian Lakes, Giżycko, Poland

^e^ Siauliai Chamber of Commerce, Industry and Crafts, Siauliai, Lithuania

^f^ Institute of Food Safety, Animal Health and Environment “BIOR”, Riga, Latvia

***^g^*** Region Norrbotten, Gällivare, Sweden

_______________________________________

^*^Corresponding author: [ewa.babkiewicz@cnbc.uw.edu.pl](mailto:ewa.babkiewicz@cnbc.uw.edu.pl)

**The following is included as supporting information for this paper:**

Number of pages: 5

Number of tables: 3

Number of figures: 2

**Table S1.** Characteristics of studied lakes. **For each lake and for each season (spring, summer, autumn), three surface-water samples (one per zone)** were collected from the same three ecologically distinct zones: (1) deepest central basin, (2) coastal zone, and (3) site adjacent to a potential point source (e.g., hotel pier, marina, drainage outlet). Sediment samples specifically covered these ecologically distinct and functionally relevant zones to capture spatial variability in sediment deposition and contamination and were sampled once in 2023 to provide a snapshot of accumulated MP deposition over the year.

| **Lake name** | **Łabap** | **Jagodne** | **Krzywa**  **Kuta** | **Mastis** | **Germantas** | **Lukstas** | **Ludza** | **Stâmerienas** | **Galgauskas** | **Pintela** |
| --- | --- | --- | --- | --- | --- | --- | --- | --- | --- | --- |
|  |  |  |  |  |  |  |  |  |  |  |
| **Country** | Poland | Poland | Poland | Lithuania | Lithuania | Lithuania | Latvia | Latvia | Latvia | Latvia |
| **Area (km^2^)** | 34.8 | 8.7 | 1.3 | 2.7 | 1.7 | 9.9 | 2.8 | 0.9 | 0.3 | 0.7 |
| **Mean depth (m)** | 7.80 | 8.7 | 6.0 | 2.6 | 2.4 | 3.6 | 4.0 | 6.5 | 2.3 | 4.1 |
| **Max depth (m)** | 13.4 | 37.4 | 26.5 | 5.3 | 12.8 | 7.0 | 11.0 | 18.7 | 4.2 | 12.0 |
| **Shoreline length (km)** | 5.4 | 35.4 | 9.4 | 12.8 | 7.1 | 20.5 | 8.67 | 4.3 | 2.6 | 5.1 |
| **Trophic status** | eutrophic | eutrophic | mesotrophic | eutrophic | oligotrophic | mesotrophic | eutrophic | eutrophic | mesotrophic | dystrophic |
| **Inflow and outflow** | present | present | present | present | present | present | present | present | present | present |
| **Population density (ind./km˛)** | 1500-2500 | 500-1500 | < 500 | 1500-2500 | < 500 | < 500 | 500-1500 | < 500 | < 500 | 500-1500 |
| **Tourism activity** | high^3^ | medium^2^ | low^1^ | high^3^ | low^1^ | medium^2^ | high^3^ | high^3^ | low^1^ | low^1^ |
| **Coordinates** | 54°7'12" N 21°38'24" E | 53°55'12" N  21°42'36" E | 54°6'0" N 21°57'36" E | 55°58'5" N 22°15'12" E | 55°58'43" N 22°8'35" E | 55°42'21" N 22°19'42" E | 57°16'10" N 26°52'36" E | 57°13'30" N 26°53'27" E | 57°13'30" N 26°31'58" E | 57°16'24" N 26°44'5" E |
| **(S) study lake**  **(R) reference lake** | S | S | R | S | R | S | S | S | R | R |
| **SUI index** | 2.4 | 16.1 | 1.1 | 29.3 | 1.5 | 0 | 2.7 | 6.1 | 0 | 0 |

low^1^ – Low number of inhabitants, few or no points of economic activity (restaurants, hotels).

medium^2^ – A medium number of inhabitants, a large number of points of economic activity (restaurants, hotels), some infrastructure for freshwater navigation, and urban beaches.

high^3^ – A high number of inhabitants, a large number of points of economic activity (restaurants, hotels), an extensive freshwater shipping infrastructure and urban beaches.

**Table S2**. Treatment applied for surface water and sediment MP samples.

| **Samples** | **Treatment** **steps** | | | | | | | |
| --- | --- | --- | --- | --- | --- | --- | --- | --- |
|  | Freeze  -drying | 30% H_2_O_2_ | Density separation I | 5% SDS | Enzymes in TRIS | Enzymes in acetate | Fenton  reaction | Density separation II |
| **Water** | **-** | **×** | **-** | **-** | **-** | **-** | **-** | **-** |
| **Sediments** | **×** | **×** | **×** | **×** | **×** | **×** | **×** | **×** |

**Table S3.** Overview of statistical testing results based on individual factor analysis.

|  |  | **Water surface** | | | | **Sediments** | | | | |
| --- | --- | --- | --- | --- | --- | --- | --- | --- | --- | --- |
| **Dependent**  **variable** |  | **Independent variables (factors)** | | | | | | | | |
|  | **Data mode** | **Country** | **Lake** | **L_type_** | **Season** | | **Country** | **Lake** | **L_type_** | **S_point_** |
| **MP_conc_** | Absolute | ns | ns | ns | ns | | ns | ns | ns | ns |
| **MP_size_** | Proportions | **5-20 mm*** | ns | ns | ns | | **<1 mm***  **1-5 mm*** | ns | ns | **<1 mm** |
|  | Absolute | ns | ns | **<1 mm*** | ns | | ns | ns | ns | ns |
| **MP_shape_** | Proportions | ns | ns | ns | **Fiber***  **Foam*** | | ns | ns | ns | ns |
|  | Absolute | ns | - | ns | **Foam*** | | ns | ns | ns | ns |
| **MP_color_** | Proportions | **Blue**  **Brown**  **Multicolored** | ns | ns | ns | | ns | - | ns | - |
| **MP_species_** | Absolute | **PP** | ns | ns | ns | | - | - | - | - |

***** Significant differences also found and confirmed in *post-hoc* analysis pairwise testing or Wilcoxon rank-sum test

**–** Not enough data for statistical analysis

**ns** No significant differences found between groups


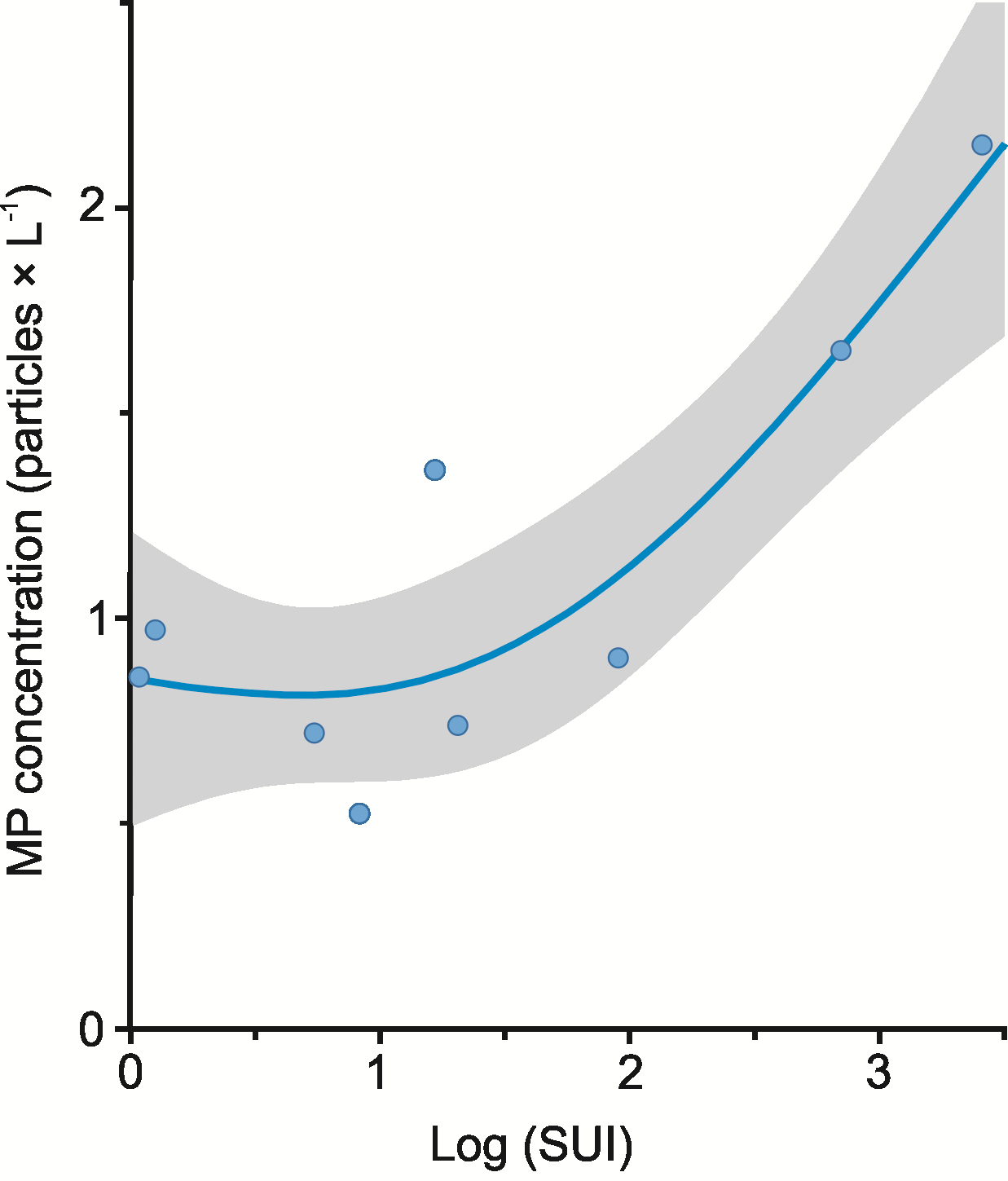


**Fig. S1.** GAM model fit (Stats1) explaining the effect of SUI on the abundance of MP particles in surface water. Black dots represent the mean values for each lake during the study period.
